# Specialty preferences among final year medical students in medical schools of southeast Nigeria: need for career guidance

**DOI:** 10.1186/s12909-016-0781-3

**Published:** 2016-10-04

**Authors:** Edmund Ndudi Ossai, Kenechi Anderson Uwakwe, Uchenna Chidi Anyanwagu, Ntat Charles Ibiok, Benedict Ndubueze Azuogu, Ngozi Ekeke

**Affiliations:** 1 Department of Community Medicine, College of Health Sciences, Ebonyi State University Abakaliki, Abakaliki, Nigeria; 2 Department of Community Medicine, College of Medicine, Imo State University Owerri, Owerri, Nigeria; 3 Department of Community Medicine, Nnamdi Azikiwe University Teaching Hospital Nnewi, Nnewi, Nigeria; 4 Epidemiology and Public Health Division, School of Medicine, University of Nottingham, Nottingham, UK; 5 Department of Community Medicine, University of Nigeria Teaching Hospital Ituku-Ozalla, Enugu, Nigeria; 6 German Leprosy and TB Relief Association Enugu, Enugu, Nigeria

## Abstract

**Background:**

In resource-poor settings with low doctor-population ratio, there is need for equitable distribution of healthcare workforce. The specialty preferences of medical students determine the future composition of physician workforce hence its relevance in career guidance, healthcare planning and policy formulation. This study was aimed at determining the specialty preferences of final year medical students in medical schools of southeast Nigeria, the gender differences in choice of specialty and the availability of career guidance to the students during the period of training.

**Methods:**

A descriptive cross-sectional study was conducted among final year medical students in the six accredited medical schools in southeast Nigeria using self-administered semi-structured questionnaire. Information on reason for studying Medicine, specialty preference and career guidance were obtained. Chi-square test of statistical significance was used in the analysis.

**Results:**

A total of 457 students participated in the study with a response rate of 86.7 %. The mean age was 25.5 ± 2.9 years and 57.1 % were male. Majority (51 %) opted to study Medicine in-order to save lives while 89.5 % intended to pursue postgraduate medical training. A higher proportion (51.8 %) made the decision during the period of clinical rotation. The five most preferred specialties among the students were Surgery (24.0 %); Paediatrics (18.8 %); Obstetrics and Gynaecology (15.6 %); Internal Medicine (11.0 %) and Community Medicine (6.8 %) while Pathology (2.0 %); Anaesthesia (0.7 %) and Ear, Nose and Throat (0.2 %), were the least preferred. Compared to females, a higher proportion of male students intended to specialise in Surgery (32.3 % vs 13.0 %, *p* < 0.001) in contrast to Paediatrics (11.2 % vs 28.8 %, *p* < 0.001). Majority of the students, 74.6 % had no form of career guidance during their stay in medical school and 11.2 % were undecided on choice of specialty.

**Conclusion:**

In spite of the high proportion of students willing to pursue specialist medical training after graduation, most opted for the four core clinical specialities of Surgery, Paediatrics, Obstetrics and Gynaecology and Internal Medicine. Majority of the students made these decisions during clinical rotations. Also, majority had no form of career guidance throughout their stay in medical school. To ensure an equitable distribution of a limited physician workforce in a resource-poor setting, there is need for proper career guidance for the students and this should be in line with the national health needs.

**Electronic supplementary material:**

The online version of this article (doi:10.1186/s12909-016-0781-3) contains supplementary material, which is available to authorized users.

## Background

As populations grow and new health needs emerge, health systems globally are faced with the challenge of meeting these needs. Occasionally there is the emergence of new diseases and changes in the trend/ pattern of existing ones. This scenario is made worse by the limited number of medical personnel and their non-uniform distribution both in areas of specialty and geographical location [1]. This has made it imperative for an equitable distribution of medical personnel in line with local health needs. {Globally the distribution of physician specialization is attracting greater attention based on the fact that the outcome of these choices may not meet community needs [2]. There is evidence that some of those applying to study Medicine already have certain specialty preferences before the commencement of studies [3]. However it has also been found that medical students may modify their attitudes towards different specialties as they pass through the various clinical rotations [4].

In Nigeria, there are 4 doctors per 10, 000 population. This is low when compared to that in many developed countries like the United Kingdom which has a ratio of approximately 30 doctors per 10, 000 people [5]. Among the six regions of the World Health Organization, the African region has the least doctor-patient ratio of 2.5/10,000 population and this is more pronounced when it is compared with the American and European regions (20.4/10,000 and 33.3/10,000 population respectively) [6]. This low doctor population ratio contributed to the critical shortage of healthcare workers in sub-Saharan Africa, including Nigeria [7]. Furthermore, the continuous exit of medical doctors from Nigeria to the developed countries in search of better opportunities has added another strain to the health sector, making it necessary for attention to be paid on career choices of medical students.

Nigeria is regarded as having one of the largest concentration of human resources for health in Africa [8]. The country has a total of 37 medical schools of which 31 have full accreditation for the training of medical doctors at the undergraduate level [9]. There are also two officially recognized institutions for the training of specialist doctors namely the West African Postgraduate Medical College and the National Postgraduate Medical College of Nigeria. It is expected that an effective management of these institutions could enable Nigeria make up for these shortages in the number of medical personnel. The quest for postgraduate medical training cannot be overemphasized especially for a developing country like Nigeria. With the emergence of new diseases and changing patterns of existing ones coupled with improved technologies and their applications to medical practice, no country could afford to lag behind in protecting the health of its citizens. This era of globalization has also raised to new heights the standards of medical practice necessitating the need for specializations and sub-specializations in Medicine.

The National Postgraduate Medical College of Nigeria is wholly indigenous and was established on 24th September 1979 about four years after the inauguration of the first College of the West African Postgraduate Medical College. The National Postgraduate Medical College has at the end of the year 2013, produced a total of 3286 medical consultants by residency training. Approximately 55 % of these consultants belong to the four core clinical disciplines of Surgery, Medicine, Paediatrics and Obstetrics and Gynaecology [10]. Also, while Obstetrics and Gynaecology has the highest number of consultants produced so far (15 %), the Ear, Nose and Throat specialty has the least (2 %) [10]. The disparities in the number of different consultants produced by the National Postgraduate Medical College reveal that some specialties maybe on the priority list of the various training centres designated for residency training in the country [11].

It is widely known that the choice of specialties for newly graduated doctors varies from country to country making it possible that each country has its own mix of specialist doctors. Undoubtedly, the specialty preferences of medical students determine the future composition of the physician workforce [12]. Hence in order to tailor this mix to national health needs, there is need for regular surveys among medical graduates to establish future needs and career opportunities [13]. This is informed by the finding that a greater proportion of medical students make their decisions on their areas of specialisation in the final year of undergraduate medical training [14] Determining the specialty preferences of medical students are also very relevant in the planning of health services [12], and in career counselling and policy formulations [15]. This study was designed to determine the specialty preferences among final year medical students in medical schools of southeast Nigeria, the gender differences in choice of specialty and the availability of career guidance to the students during the training period.

## Methods

### Setting

The study was conducted in medical schools in southeast Nigeria, which is one of the six geo-political zones of Nigeria. It is made up of five states including Abia, Imo, Ebonyi, Anambra and Enugu states. The southeast zone of Nigeria has a population of 16, 381, 729 people based on the 2006 national population census [16], and lies within a total area of 28, 987 km^2^ [17]. The inhabitants are mostly of Igbo ethnic nationality and are predominantly Christians.

The southeast zone of Nigeria has sixteen universities comprising of five federal and five state universities while the remaining six are privately owned. Medicine is accredited for study in six universities in the zone and all the six medical schools were included in this study. This is because the accreditation exercise usually conducted by the National Universities Commission gives the various medical schools the legal rights to train medical students who will make tomorrow’s healthcare workforce. Two of these universities belong to the Federal Government of Nigeria and they include Nnamdi Azikiwe University Awka, and the University of Nigeria Nsukka, which was established in 1960, and is Nigeria’s second oldest university. The state owned universities that offer Medicine in the zone include those of Abia, Imo, Ebonyi and Enugu states.

### Study design

The study employed a descriptive cross-sectional study design.

### Study participants

The study population consisted of all final year medical students in medical schools of southeast Nigeria who gave consent to participate in the study. A total of 457 final year medical students in the six medical schools participated in the study representing a response rate of 86.7 %. The participating medical schools included University of Nigeria Nsukka, (132 medical students, response rate 95 %), Nnamdi Azikiwe University Awka, (79 students, response rate 82.3 %), Abia State University Uturu, (67 students response rate 80.7 %), Ebonyi State University Abakaliki, (79 students, response rate 90.8 %), Enugu State University of Science and Technology Enugu, (37 students, response rate 74 %) and Imo State University Owerri, (63 students, response rate 87.5 %).

### Study instrument

The study instrument was a pre-tested, semi-structured questionnaire which was self administered. The questionnaire was designed by the researchers and had thirty variables (with relevant subunits) and included open and close ended questions based on validity following the pre-test. The data for reasons in the questionnaire were collected using open ended questions and the individual responses were organized into themes. Information was obtained on the socio-demographic characteristics of the students, reason for studying Medicine, intention to pursue postgraduate medical training, specialty of choice, when decision on specialization/ choice of specialty was made and whether there was any form of career guidance during the period of training in medical school.

### Data analysis

The analysis was performed using Statistical Package for Social Sciences (SPSS), statistical software version 20. Frequency tables and cross tabulations were generated. Chi square test of statistical significance was used in the analysis and level of significance was based on a *p*-value of less than 0.05.

## Results

The mean age of the students was 25.5 ± 2.9 years and the highest proportion of the students (51.6 %) were in the age group 25–29 years followed by those who were less than 24 years old, (41.6 %). Those more than 34 years old formed the least proportion, (1.8 %). A higher proportion of the students, (57.1 %) were male while majority, (95.8 %) were of Igbo ethnic nationality. Also a minor proportion of the students, (9.4 %) were married and a higher proportion (53.6 %) were students of the various state owned universities.

Table 1, shows the reasons for studying Medicine by the students.. Majority of the students, (51 %), decided to study Medicine in order to save lives and serve humanity. A minor proportion of the students, (4.6 %) were studying Medicine based on their childhood dreams.

**Table 1:** The reason for studying Medicine by the students

Variable	*n* = 457 (Frequency)	Percent (%)
Reason for studying medicine		
Save lives/service to humanity	233	51.0
Personal interest	90	19.7
Prestige/finance	62	13.6
Influenced by others	22	4.8
Childhood dream	21	4.6
No specific reason	29	6.3

Table 2, shows the preferred specialty and specialty of choice of the students for postgraduate training. A higher proportion of the students (89.5 %), intended to pursue specialist training and majority (51.8 %), made the decision during the period of clinical rotation. Obstetrics and Gynaecology was regarded by the students as the most preferred specialty during the clinical rotations while Surgery was first in the specialty of choice for postgraduate training.

**Table 2:** Preferred specialty and specialty of choice for postgraduate training

Variable	*n* = 457 (Frequency)	Percent (%)
Intention to specialize after graduation		
Yes	409	89.5
No	48	10.5
When decision was taken	*n* = 409	
Before entry to medical school	130	31.8
During preclinical period	67	16.4
During clinical period	212	51.8
Most preferred specialty during clinical rotation		
Obstetrics & Gynaecology	144	31.5
Surgery	88	19.3
Paediatrics	60	13.1
Internal Medicine	44	9.8
Community Medicine	30	6.6
Psychiatry	28	6.1
Ophthalmology	19	4.2
Radiology	10	2.2
Pathology	4	0.9
Anaesthesia	1	0.2
No specific specialty	29	6.3
Specialty of choice of the students for postgraduate medical training		
Surgery	98	24.0
Paediatrics	77	18.8
Obstetrics and Gynaecology	64	15.6
Internal Medicine	45	11.0
Community Medicine	28	6.8
Ophthalmology	15	3.7
Psychiatry	14	3.4
Radiology	10	2.4
Pathology	8	2.0
Anaesthesia	3	0.7
Ear, Nose and Throat	1	0.2
Undecided	46	11.2

Table 3, shows the reasons for preferred specialty during clinical rotations and specialty of choice for postgraduate training. The major reason why Obstetrics and Gynaecology was the most preferred specialty by the students was related to the lecturers, (that they were friendly, offered good advice and the students had good interactions with them). Also, the major reason why Surgery was the specialty of choice for postgraduate training was personal interest.

**Table 3:** Reasons for preferred specialty and specialty of choice for postgraduate training

Variable	*n* = 457 (Frequency)	Percent (%)
Reason Obstetrics & Gynaecology was most preferred	*n* = 144	
Lecturer related factors^a^	44	30.6
Department is organized	41	28.5
Personal Interest	33	22.9
Interest in Women/delivery services	19	13.2
No specific reason	7	4.9
Reasons for choosing Surgery for postgraduate medical training	*n* = 98	
Personal interest	57	58.2
Relevance of services	24	24.5
Finance	6	6.1
Influenced by others	4	4.1
No specific reason	7	7.1

^a^lecturers friendly, offered good advice and related well with students

Table 4, shows the least preferred specialty and reasons. Community Medicine was the least preferred specialty by the students during the clinical rotations and the major reason for that was that the specialty was non clinical hence regarded as uninteresting.

**Table 4:** Least preferred specialty and reasons

Variable	*n* = 457 (Frequency)	Percent (%)
Least preferred specialty during clinical rotation		
Community Medicine	94	20.6
Internal Medicine	60	13.1
Paediatrics	45	9.8
Pathology	33	7.2
Psychiatry	31	6.8
Ear, Nose & Throat	29	6.3
Surgery	29	6.3
Obstetrics and Gynaecology	29	6.3
Radiology	13	2.8
Anaesthesia	13	2.8
No specific specialty	81	17.7
Reason Community Medicine was least preferred	*n* = 94	
Uninteresting/non clinical	37	39.4
Poor guidance/lecturers attitude	26	27.7
Not challenging	20	21.3
Stressful	4	4.3
No specific reason	7	7.4

Table 5, shows career guidance for the students. There was no form of institutional career guidance for the students. However a minor proportion of the students, (25.4 %) received some form of career guidance. Ebonyi State University had the highest proportion of students (36.7 %) who received career guidance while Imo State University had the least proportion, (17.5 %).

**Table 5:** Career guidance for the students

Variable	*n* = 457 (Frequency)	Percent (%)
Provision of career guidance by the Institution		
No	457	100
Received some form of Career guidance		
Yes	116	25.4
No	341	74.6
Students that received some form of career guidance based on Institution*	*n* = 116	
Abia State University	21	31.3
Ebonyi State University	29	36.7
Enugu State University	13	35.1
Imo State University	11	17.5
Nnamdi Azikiwe University	18	22.8
University of Nigeria Nsukka	24	18.2

*P value on bivariate analysis = 0.013

Table 6, shows the gender differences on specialty of choice of students. A higher proportion of male students intend to specialize in Surgery when compared with the female students and the difference in proportions was found to be statistically significant, (*p* < 0.001). Also, a significantly higher proportion of female students intend to specialize in Paediatrics when compared with their male counterparts (*p* < 0.001).

**Table 6:** Gender differences on specialty of choice of students

Specialty	Male	Female	Total	*P value
	*n* = 232	*n* = 177	*n* = 409	
	N (%)	N (%)	N (%)	
Surgery	75 (32.3)	23 (13.0)	98 (24.0)	<0.001
Paediatrics	26 (11.2)	51 (28.8)	77 (18.8)	<0.001
Obstetrics and Gynaecology	43 (18.5)	21 (11.9)	64 (15.6)	0.066
Internal Medicine	26 (11.2)	19 (10.7)	45 (11.0)	0.880
Community Medicine	13 (5.6)	15 (8.5)	28 (6.8)	0.255
Ophthalmology	5 (2.2)	10 (5.6)	15 (3.7)	0.062
Psychiatry	8 (3.4)	6 (3.4)	14 (3.4)	0.974
Radiology	5 (2.2)	5 (2.8)	10 (2.4)	0.664
Pathology	7 (3.0)	1 (0.6)	8 (2.0)	0.076
Anaesthesia	1 (0.4)	2 (1.1)	3 (0.7)	0.412
Ear, Nose and Throat	0 (0.0)	1 (0.6)	1 (0.2)	0.252
Undecided	23 (9.9)	23 (13.0)	46 (11.2)	0.329

*p value on bivariate analysis

## Discussion

Majority of the students (51 %), in this study opted to study Medicine because of their intention to save lives and serve humanity. This is commendable as it is of societal good however this result is at variance with one from a study in South Africa, where the major reason for studying Medicine among the students was personal interest [18]. The different reasons for studying Medicine by the students in the two countries could be attributed to the background differences of the students. Also, these differences in the intentions of students for studying Medicine may have a role to play in the specialty of choice of these medical students. However what remains open to policy makers in these countries is how to harmonize these various intentions of the students for studying Medicine for the good of the country and its people.

The students’ overall perception of Obstetrics and Gynaecology is a good report of the lecturers in that specialty in all the study institutions. Also, it further supports the finding that innovativeness and dedication on the part of teachers could arouse interest of students in a particular specialty during the clinical rotations [19]. Similarly the students’ poor perception of Community Medicine could be explained by the fact that direct patient care has remained the main professional interest of medical students [20] and this is not the focus of this branch of Medicine. However this specialty, Community Medicine has been described by an eminent scholar as one that took a very long time to emerge in Nigeria and which has been having problems in determining its scope and area of practice even among the practitioners [10]. Based on this observation, the opinion of the students about Community Medicine is not surprising, however since every medical specialty has its own uniqueness, the practitioners in this field only need to look inwards and find some aspects of Community Medicine that will help to resolve this internal conflict and introduce the specialty properly to the students and those outside the medical sphere.

Majority of the students (89.5 %), preferred to pursue specialist training after graduation from medical school. With the continuous improvements in medical practice, specialization in Medicine has become the global trend as similar high proportion of medical students were willing to pursue specialist training from the results of several studies [18, 19, 21–24]. In Nigeria, this trend of increasing quest for specialization by medical doctors has been partly attributed to improved remunerations and work conditions in public tertiary health institutions where these trainings take place [24]. The high proportion of medical students in Nigeria seeking specialist training also has policy implications as these tertiary health institutions should be adequately prepared for increased intake of medical graduates for postgraduate medical training. There is every need for a workable plan to progressively increase the number of spaces for placement of Resident Doctors of different specialties in the various tertiary health institutions and other centres designated for postgraduate medical training.

An approximate one third of the students that participated in this study had concluded on specialization and the specialty of choice before gaining admission into the various universities to study Medicine. This is similar to results of two studies among Croatian medical students [25, 26] and further confirms the observation that some medical students already have a choice of medical specialty for specialization before enrolment in medical schools [3]. It is possible that this group of students may have been influenced by personal convictions or family members. The research finding that recognizes practical experience within a particular specialty as an important factor in the choice of specialty of students [19, 27] could have been responsible for the outcome that the majority of the students (51.8 %), made the decision on choice of specialty during the clinical rotations. This could be attributed to the close interactions between the students and their teachers who are medical consultants in the various specialties of Medicine and who most times serve as mentors for the students during and after the training period.

The specialty preferences of the students were centred on the four broad clinical specialties of medical practice which included Surgery, Paediatrics, Obstetrics and Gynaecology and Internal Medicine as reflected in the high proportion of the students (62 %) who indicated their interest in these four specialties. This is similar to results of previous studies in Nigeria [19, 24, 28, 29], It appears that the overall picture of medical practice in Nigeria is still anchored on these core clinical specialties which are the focus of medical internship in Nigeria and also the ones with the longest duration of student clerkship during the undergraduate training period. It has also been observed that placement of medical graduates for postgraduate medical training in tertiary health institutions in Nigeria is tilted in favour of these four clinical specialties [11].

A minor proportion (9.4 %) of the respondents opted to specialize in five specialties including Psychiatry, Radiology, Anaesthesia, Ophthalmology and Ear, Nose and Throat. In the training of medical doctors in Nigeria, these specialties are regarded as minor postings with relatively shorter periods of clerkship. The low proportion of students interested in these aspects of Medicine could be because of the short duration of postings, the overbearing influence of the four core clinical specialties on the students or that the practitioners in these fields have not aroused the sensibilities of the students to these specialties of Medicine. The proportion of students who were interested in these five specialties of Medicine was less than the proportion that were still undecided on specialty of choice few months to graduation.

None of the students indicated interest in pursuing a career in the three basic medical sciences of Biochemistry, Anatomy and Physiology which form the basis of pre-clinical medical studies. This could be because the students may have all their attention focused on direct medical care of patients. Also, none of the students had the intention to specialize in Family Medicine. An explanation for this could be that there has been no direct contact of medical students with Family Physicians in Nigeria during the period of medical training up to the time of this study. Also, of the six teaching hospitals in southeast Nigeria where these students receive clinical training only one is an accredited centre for training of Family Physicians at the postgraduate level. It has been found that medical specialties in an institution coupled with practical experiences in that specialty could influence the interest of students in that specialty [27]. Bearing in mind the relevance of Family Physicians in the healthcare delivery system of any country, it is hoped that this will change following the current involvement of Family Physicians in the training of medical students in Nigeria as there will be close clinical interactions between medical students and Consultant Family Physicians.

The most preferred specialty of choice among the students for postgraduate training was Surgery and this has remained a consistent finding especially in Africa [19, 21, 23, 24, 28–32]. It could be that Surgery as a specialty (including all its sub-divisions like Orthopaedics, Urology, Plastic and General Surgery etc.) has stood firm as one of the chief specialties of Medicine over the years and this has continued even to the detriment of other surgery related subspecialties. From the results of this study, the ratio of the students interested in specializing in Surgery when compared to Anaesthesia was 33 to 1. For the students whose interest was to specialize in Surgery, the major reason for the decision was personal interest. This concept of personal interest has long been identified as the major factor in choice of specialties by medical students [28, 30, 33–35].

There were also observed gender differences in choice of specialty from the results of this study and this was pronounced for Surgery and Paediatrics. A significantly higher proportion of male students preferred to specialize in Surgery when compared to female students. The reverse was the case for female students as it concerned Paediatrics. This could mean that Surgery could be male dominated while Paediatricians may have more of female practitioners in the study area. Similar results were obtained from studies in Kenya [21] and Germany [36]. However, from a study in Sweden [37] and Saudi Arabia [38], a significantly higher proportion of female medical students had the intention to specialize in Gynaecology when compared with their male counterparts. This supports the idea that choice of specialty for medical doctors vary from country to country.

An important finding from this study is that in the six medical schools in southeast Nigeria, there was no form of institutional career guidance for the students. This may possibly be the situation in the other medical schools in Nigeria. Also, a higher proportion of the students, (74.6 %) had no form of career guidance whatsoever throughout their stay in the medical school. Variations exist in the proportion of medical students that received career guidance in Africa. From a study in South Africa, it was 54 % [18], while a study in Gambia reported as high as 90 % of medical students having no form of career counselling [39]. Perhaps the absence of career counselling among the respondents in this study may be the reason why 11.2 % of the students were yet to make a decision on their specialty of choice few months to graduation. This proportion of students may eventually be swayed by personal interest or influenced by family members and peers on choice of specialty. Higher proportions of students than that reported in this study were undecided on choice of specialty for postgraduate medical training from the results of other studies in Africa [21, 23]. Absence of career guidance for medical students at this stage of our development in Nigeria may be a pointer that there is no health manpower need assessment and consequent future projections in terms of the country’s need of the various medical specialties which may be an indication of poor planning of health services in the country.

It has been observed that the career plans of medical students in Africa are not aligned with the health manpower needs of the continent [23] and this may eventually have effects on the health needs of communities [2]. This of necessity calls for career education and counselling for medical students in medical schools in Nigeria and other African countries. This is because a well-coordinated career education and counselling will assist the students to realize their full potentials in the profession by reason of right choices [40]. Also, when initiated on time it could ensure that medical students especially those who are unsure of choice of specialty are guided to specialties in which there are manpower shortages [41].

There has been a call for interest in career education and counselling of medical students in Nigeria [28], same also in Kenya [21]. In Gambia, all the medical students that participated in a study expressed interest in having career counselling during the period of their training [39], and from a similar study in Israel came the call for greater interest in the career selection of medical students [42]. The same study also earmarked the fifth year in medical school as the appropriate time for guidance to medical students on choice of specialty [42]. This was also highlighted in our study as more than half of the students (51.8 %) made their choice of specialty for specialization at that stage of their clinical training. Career education and counselling for medical students will help fine tune the aspirations of medical students which is mostly determined by personal interest to fall in line with the national health manpower need thus ensuring that the country has the correct mix of specialist doctors able to face its own peculiar health challenges. Based on these observations, there is every need to institutionalize career guidance and counselling in medical schools in Nigeria.

## Conclusions

Majority of the students in the study area opted for postgraduate medical training after graduation and their choice of specialties centred around the four core clinical specialties of Surgery, Paediatrics, Obstetrics and Gynaecology and Internal Medicine. Majority of the students decided on specialization and specialty of choice during the clinical rotation period. Also, majority of the students had no form of career guidance throughout their stay in the medical school. There is need for career guidance for the students and this should be in line with the national health needs.

## Additional file

Additional file 1: Questionnaire. Description: Questionnaire for Specialty preferences among final year medical students in medical schools of southeast Nigeria: need for career guidance as designed by the researchers. (DOCX 23 kb)
