# Supplementary material for: Specialty preferences among final year medical students in medical schools of southeast Nigeria: need for career guidance
Source: BMC Med Educ. 2016 Oct 4;16:259. doi: 10.1186/s12909-016-0781-3 (PMC5050581; doi:10.1186/s12909-016-0781-3)
Supplement: Additional file 1: — Questionnaire. Description: Questionnaire for Specialty preferences among final year medical students in medical schools of southeast Nigeria: need for career guidance as designed by the researchers. (DOCX 23 kb) [file 12909_2016_781_MOESM1_ESM.docx]

**QUESTIONNAIRE**

**SPECIALTY PREFERENCES AMONG FINAL YEAR MEDICAL STUDENTS IN MEDICAL SCHOOLS IN SOUTH-EAST NIGERIA**

**Introduction**

*Socio-demographic characteristics*

1. Age **(last birthday)** ……………………………………….
2. Gender

A. Male ( )

B. Female ( )

1. Ethnic group

A. Ibo. ( )

B. Yoruba ( )

C. Hausa ( )

D. Ijaw ( )

E. Others specify ……………………………………………

1. Marital status.

A. Single ( )

B. Married ( )

C. Others, specify ………………….

1. Religion.

A. Christianity ( )

B. Muslim ( )

C. Traditional African Religion ( )

D. Others specify………………..

1. Father’s highest Education

A. No formal education ( )

B. Primary education ( )

C. Secondary education ( )

D. Post secondary education ( )

E. Others specify ……………………….

1. Mother’s highest Education

A. No formal education ( )

B. Primary education ( )

C. Secondary education ( )

D. Post secondary education ( )

E. Others specify ……………………….

1. Occupation (**Father)** ………………………………………………

1. Occupation (**Mother**) ……………………………………………….
2. Place of Family Residence. (state name of town, community or village …………………………………………………….

*Specialty Preference*

1. Name of Secondary school attended, ( please include the name of the town the school is located) ………………………………………………………………………………………………………………………………………………………………………………………………
2. Year of graduation from Secondary School. ………………………………………
3. a) Did you have any work experience before entering Medical School.

A.Yes ( )

B.No ( )

1. If Yes, indicate the number of years.. ……………………………..
2. Who is paying your school fees in the Medical School. …………………………………………………………………………………….
3. What is your **major reason** for studying Medicine …………………………………………………………………………………………. …………………………………………………………………………………………..
4. a). If given another opportunity, will you still study Medicine

A. Yes ( )

B. No ( )

C. Don’t know ( )

1. What is your reason for Yes or No or Don’t know above

………………………………………………………………………………………………………………………………………………………………………………………………

c). If No, What will you prefer to study ……………………………………

1. a). Having rotated through all the departments, which Department /Specialty was the most preferred to you. ……………………………………………………………………………

b). What made the Department/Specialty above most preferred ………………………………………………………………………………………………………………………………………………………………………………………………

1. a). What Department/ Specialty was the least preferred to you …………………………………………………………………………………………..

b). What made the Department/ Specialty above the least preferred ………………………………………………………………………………………………………………………………………………………………………………………………

1. a). Do you intend to pursue Specialist Training (Residency Programme) after graduation

A. Yes ( )

B. No ( )

C. Not yet Sure ( )

b). What is your reason for the Yes, No or Not yet Sure above ………………………………………………………………………………………………………………………………………………………………………………………………

1. If Yes, when was the decision made. ………………………………………………………………………………………………………………………………………………………………………………………………
2. What specialty do you intend to specialize in ………………………………………………………………………
3. What is your **major reason** for choosing the Specialty ………………………………………………………………………………………. ……………………………………………………………………………………….
4. a). Which country do you intend to pursue the Specialist/ Residency training
5. Nigeria (pls specify the Institution) …………………………………………………..

B. Other African country (pls specify the country) …………………………….. …………………………………………………………………………..

C. Outside Africa (pls specify country) ……………...................................................

b). What is your reason for choosing the country above. ………………………………………………………………………………………………………………………………………………………………………………………………

.

1. a). Which sector will you prefer to practice if you do **NOT** intend to pursue Residency/ Specialist training.

A. Own a Private Hospital ( )

B. Work in Government Hospital ( )

C. Work in a University ( )

D. Work in a Private Hospital ( )

E. Non Governmental Organization ( )

F. Work for Government and own a Private Hospital ( )

G. Others (specify). ……………………………………………………

b). What is the reason for your answer above. ………………………………………………………………………………………………………………………………………………………………………………………………………………………………………………………………………………..

1. a). If you are **NOT** going to pursue Specialist /Residency Training, Where do you intend to practice.
2. Nigeria (please specify the City or Town) …………………………………………………..

B. Other African country (please specify the country) …………………………….. …………………………………………………………………………..

C. Outside Africa (please specify country) ……………...................................................

b). What is your reason for choosing the country above. ……………………………………………………………………………………………..

1. Did you receive any form of **career guidance** while in the Medical School.

A. Yes ( )

B. No ( )

C. Not Sure ( )

1. How do you view your future after graduation from the medical school.

A. Bright ( )

B. Not Bright ( )

C. Uncertain ( )

D. Not Sure ( )

1. What are your expectations after graduation. ………………………………………………………………………………………………………………………………………………………………………………………………

Thank you.
